# Factors influencing the success of targeted weight loss in healthcare providers with overweight and obesity after a six-month weight reduction intervention program

**DOI:** 10.1371/journal.pone.0330018

**Published:** 2025-08-18

**Authors:** Siti Fatimah Samsury, Mohd Nazri Shafei, Mohd Ismail Ibrahim, Wan Nor Arifin Wan Mansor, Noriah Mahmud

**Affiliations:** 1 Department of Community Medicine, School of Medical Sciences, Health Campus, Universiti Sains Malaysia, Kota Bharu, Kelantan, Malaysia; 2 Unit of Biostatistics and Research Methodology, School of Medical Sciences, Health Campus, Universiti Sains Malaysia, Kota Bharu, Kelantan, Malaysia; 3 Environmental Health Unit, Terengganu State Health Department, Wisma Persekutuan, Kuala Terengganu, Terengganu, Malaysia; UCMI: University College MAIWP International, MALAYSIA

## Abstract

Obesity among healthcare providers (HCPs) affects their health and ability to provide care. In Terengganu, Malaysia, where 34% of adults are obese and over 50% of the workforce is overweight, targeted interventions are needed. Despite the importance of weight management in healthcare professionals, there is a lack of research focusing on effective strategies within this specific population. The study was conducted to identify factors associated with the success of targeted weight loss in HCPs with overweight and obesity following a six-month weight reduction intervention program. A cohort study involving HCPs with a body mass index (BMI) of 25 kg/m^2^ or higher was conducted in Terengganu, Malaysia, spanning from March to October 2023. All participants were required to undergo a six-month weight reduction intervention program. A participant who achieved a weight reduction of ≥ 5.0% during the follow-up period was considered successful in reaching the targeted weight reduction. Multiple logistic regression was applied to determine the factors associated with the success in achieving the targeted weight reduction. We found that the proportion of successfully achieved targeted weight reduction was 26.0%. The factors that were associated with the success of targeted weight reduction among HCPs with overweight and obesity were income (Adjusted Odds Ratio [AOR]: 0.94; 95% Confidence Interval [CI]: 0.88, 1.00, p-value 0.039), number of programs attended (AOR: 1.34; 95% CI: 1.02, 1.75, p-value 0.035), and the frequency of calorie intake recorded (AOR: 1.05; 95% CI: 1.02, 1.08, p-value 0.001). In conclusion, the proportion of successfully achieved targeted weight reduction was relatively low. To address this issue, future interventions need to concentrate on reinforcing participant self-monitoring and commitment levels. By emphasizing active progress tracking and cultivating strong dedication to the program’s objectives, there is a potential for significant improvements in weight management outcomes.

## Introduction

The escalating global prevalence of overweight and obesity has surged to concerning levels, presenting a significant public health issue [1]. A higher body mass index (BMI) is associated with a greater risk of developing specific non-communicable diseases [2]. Furthermore, a higher BMI is associated with diminished occupational productivity, decreased levels of physical fitness and muscular strength, and an increased susceptibility to musculoskeletal pain [3–5].

The prevalence of disease and non-communicable diseases (NCD) factors among workers in Malaysia is high, with 34.0% of Malaysian adults with obesity and over half being workers [6]. Obesity among HCPs is a significant issue, as it impacts their morbidity and mortality. Research conducted in Malaysia revealed that approximately 20% of healthcare providers (HCPs) were classified as obese [7,8]. Health conditions, including obesity, significantly impact workplace performance, resulting in increased presenteeism and absenteeism among employees. Obesity may pose challenges in utilizing appropriate equipment and fulfilling the physical requirements of the job, leading to increased presenteeism and absenteeism [9]. Obesity among HCPs has been associated with presenteeism, a condition in which individuals continue working despite experiencing physical discomfort, fatigue, and chronic health issues. Research suggests that obesity-related musculoskeletal pain, metabolic conditions, and reduced endurance can impact cognitive function and workplace efficiency, leading to decreased productivity and an increased likelihood of medical errors [10].

The workplace stands as the optimal environment for health promotion and intervention targeting HCPs, as most of them spend their time in the setting. It was found that workplace wellness programs for weight reduction may enhance the health of the employees, lower absenteeism and healthcare costs, and increase their quality of life which may influence their productivity at work [11,12]. In the realm of achieving targeted weight loss, an extensive review of existing literature has pinpointed various impactful elements. These encompass age, BMI, sex, race, psychological health, individual commitment, and self-perceptions of body image [13–18].

Successful weight loss and maintenance depend on multiple factors, including caloric intake, physical activity, self-monitoring, psychological influences, social support, and structured weight loss programs [19]. While caloric intake and physical activity remain central components, research highlights that behavioral and psychological aspects are equally critical. For example, individuals with higher intrinsic motivation and structured support systems tend to achieve better weight loss outcomes and long-term adherence [19]. In addition, disordered eating patterns like binge eating, emotional eating, or night eating syndrome may also play a significant role in hindering long-term weight management among healthcare providers with obesity. Individuals with disordered eating behaviours often exhibit strong motivation to change but face physiological and psychological barriers that undermine sustained weight loss [20,21].

It was observed that older participants who underwent an internet-based intervention and received monthly personal counselling achieved greater and sustained weight reduction over three years compared to their younger counterparts [17,22]. The impact of a bodyweight reduction intervention was also found to be more pronounced in men [16,23]. Research also indicates that income may influence the likelihood of success in targeted weight reduction efforts as individuals with a higher income may have greater access to resources such as healthier food options, fitness facilities, and professional guidance [24].

The level of commitment exhibited by the participants in weight-loss programs is an important determinant of success as demonstrated that those who attended a greater number of sessions experienced a more substantial weight reduction [13]. This finding is further supported by the significant association of self-monitoring of calorie intake and physical activities using appropriate tools with higher rates of successful weight loss [23,25,26].

Weight loss interventions among healthcare workers (HCPs) in Malaysia remain underexplored. Leow et al. (2022) demonstrated that a three-month group education program with dietary counseling significantly improved weight loss outcomes among HCPs [27], while Hussain et al. (2018) reported a 46.2% success rate for hospital-based weight reduction programs, highlighting the effectiveness of structured workplace interventions [28]. However, there is a notable lack of in-depth research on the success of weight reduction programs for HCPs with overweight and obesity across diverse healthcare settings in Malaysia. Addressing this gap is critical to understanding the unique challenges and factors influencing weight management program success in this population. Therefore, this study aimed to identify factors associated with the success of targeted weight reduction among HCPs in Terengganu.

## Methods

### Study design and participants

A prospective cohort study was conducted from March to October 2023 in Terengganu, Malaysia, comprising HCPs with a BMI of 25 kg/m² or above who took part in a six-month weight management intervention utilizing the Trim and Fit 2020 Weight Management Program Module. The study excluded pregnant HCPs, those with physical limitations hindering strenuous activities, individuals who had undergone surgery in the past six months, and HCPs involved in other weight reduction programs.

The required sample size was calculated using two independent proportions formula. Assuming a proportion of success in weight loss of 30% in the intervention group and 10% in the control group [28], with a power of 80% and a 95% confidence level, the required sample size was 121. However, only 100 participants were eligible and consented to the study. The lists of HCPs with a BMI of 25 kg/m^2^ and above were obtained from the health screening records of HCPs in the districts and the state health office in Terengganu, with permission granted from the State Health Director. The participant selection process for this study employed a multistage random sampling technique. This method involved stratified sampling within five districts, followed by simple random sampling. To accomplish this, we utilised a Simple Random Sampling Generator [29] to select the participants and reach the desired sample size.

### Research tools and data collection

The intervention program was adapted from the module of trim and fit body weight management program by the Malaysian Ministry of Health [30]. The module was developed by a multidisciplinary team of dietitians, physiotherapists, and public health specialists. This program has been previously implemented in corporate and hospital settings, where it demonstrated a 42% success rate in achieving ≥5% weight loss in 3 months [27]. Specifically tailored for HCPs, this intervention incorporated flexible meal plans, stress management techniques, and structured exercise regimens that accommodated irregular working hours and shift rotations.

The Trim and Fit Weight Management Program includes three components, which comprise nutrition, physical and recreational activities, and motivation. This program includes seminars, weekly intervention activities, and monthly intervention activities. Our research tools consisted of a proforma, a calibrated digital weighing scale (Tanita BC-601, Tokyo, Japan), and a Trim and Fit Program logbook.

The participants were also required to answer the Malay version of the Body Shape Questionnaire-Short Form (BSIQ-SF) to assess their body image perception. It was a validated tool designed to measure body dissatisfaction and concerns related to body shape and weight. The BSIQ-SF consists of 21 items that evaluate feelings and behaviours associated with body image, such as preoccupation with body shape, fear of weight gain, and avoidance of body-related situations. Participants rated their agreement with each statement on a 5-point Likert scale (1 = ‘Not at all True of Myself’, 6 = ‘Completely True of Myself’). Scores for ‘Negative Affect’ and ‘Height Dissatisfaction’ ranging from 1 to 40, ‘Attractiveness Evaluation’ from 1 to 30, and ‘Physical Functionality Awareness’ from 1 to 20 with higher scores indicating greater body dissatisfaction. The scale’s internal consistency was confirmed with Cronbach’s alpha values ranging from 0.797 to 0.850, indicating a strong reliability [31].

After obtaining written consent from the participants, basic sociodemographic and work-related information was collected using proforma at the beginning of the study. Additionally, baseline physical measurements were collected including: (1) Body weight (kg), measured using a Tanita BC-601 digital scale; (2) Height (cm), measured using a stadiometer with participants standing upright without shoes; (3) Body Mass Index (BMI, kg/m²), calculated as weight (kg) divided by height (m²).

To assess the commitment of each participant to the weight management program, they were provided with the Trim and Fit program logbook, which had to be filled out daily. The logbook was divided into three sections which were Weekly Challenge Progress, Calorie Intake Record Diary, and Exercise Record Diary. Participants recorded their daily calorie consumption and calculated their own calorie intake needs. The exercise record diary tracked daily workouts, including type, duration, and intensity. Participants were taught how to calculate their calorie intake during the seminar. Their attendance was also tracked as part of the commitment assessment. Each of these variables was recorded individually as a numerical value (in number of days) to determine the participant’s commitment.

Over six months, they participated in various activities, including seminars, weekly sessions, and monthly programs, as part of the Trim and Fit Weight Management Program. Participants were assessed at baseline (Week 0), mid-intervention (Week 12), and post-intervention (Week 24). The follow-up period lasted up to 6 months (Week 48) to assess weight maintenance among participants and those who achieved significant weight loss (≥5% of baseline weight) were classified as successful [32].

### Statistical analysis

Data were entered and analysed using the IBM Statistical Program for Social Sciences (SPSS) version 28. Data was thoroughly checked and cleaned to ensure accuracy. The outcome variable was the percentage of participants achieving ≥5% weight loss at the end of the 6-month intervention. Meanwhile, the independent variables included both numerical variables (Age, income, working hours per week, attendance, dietary and exercise records, negative affect score, attractiveness evaluation score, and height dissatisfaction) and categorical variables (Gender, marital status, education level, and job position). Descriptive statistics were calculated for all variables to provide a summary of the data. For numerical variables, central tendencies, distributions, and data normality were assessed using histograms with normality plots. Categorical variables were analysed for frequency and percentage distribution. Dummy variables were created for categorical variables as needed. The last observation carried forward (LOCF) method was used to impute missing data from participants who lost to follow-up [33]. In the initial analysis, simple logistic regression was performed to identify factors associated with the success of targeted weight reduction among HCPs in Terengganu.

Crude odds ratios (OR) were derived from the simple logistic regression, and variables with a *p*-value of less than 0.25 were chosen for further analysis in multiple logistic regression analysis [34]. In the multivariable analysis, forward LR, backwards LR, and the enter methods were employed to compare models and establish the preliminary main effect model. Multicollinearity was assessed using tolerance, variance inflation factor (VIF), and the correlation matrix. Two-way interaction analyses were conducted to assess potential moderating effects on weight loss outcomes. These analyses examined interactions between income and total program attendance, income and frequency of recorded calorie intake in the logbook, and total program attendance and frequency of recorded calorie intake in the logbook. The goal was to to assess potential moderating effects on weight loss success. The fitness of the model was evaluated using the Hosmer-Lemeshow goodness of fit test, classification table, and the area under the curve, Receiver Operating Characteristic (ROC) [35]. The result of the final model was presented with an adjusted odds ratio, 95% confidence intervals (CI), Wald statistics, and *p*-values. The significance level was set at a *p-*value of less than 0.05 with two-tailed testing.

### Ethical considerations

The Human Research Ethics Committee of Universiti Sains Malaysia (USM/JEPeM/22080558) and the National Medical Research Register (NMMR) Malaysia (NMRR ID-22–02093-DWX) approved this study to be conducted. To maintain data confidentiality, participant identities were anonymized, and all data were securely stored with limited access granted solely to authorized personnel. Subsequently, all remaining data was coded to provide additional protection for participant confidentiality.

## Results

A total of 100 participants were involved in the study. The participants’ mean age was 38.8 (SD 6.40) years old. The study consisted of 17.0% males and 83.0% females. The commitment of the participants to the weight management program was assessed through attendance (days), frequency of calorie intake recorded in a logbook (days), and frequency of exercise recorded in a logbook (days). We found that the mean (SD) for attendance (days), frequency of calorie intake recorded in a logbook (days), and frequency of exercise recorded in a logbook (days) were 8.4 (3.88), 58.36 (43.46), and 63.68 (45.83) respectively. The study found that 26.0% of the participants achieved the targeted weight reduction during the 6-month study period. The characteristics of the participants (sociodemographic, commitment and body image perception score) are presented in Table 1.

**Table 1:** Characteristics of healthcare providers in Terengganu (*n *= 100).

Variables	Mean (SD)	*n* (%)
Age (years)	38.8(6.40)	
Gender		
Male		17(17.0)
Female		83(83.0)
Marital status		
Single/Window/Divorced		16(16.0)
Married		84(84.0)
Education level		
Secondary		37(37.0)
Tertiary		63(63.0)
Job positions		
Administrative Staff		13(13.0)
Nurses		44(44.0)
Other healthcare professions^a^		43(43.0)
Income	3900.7(4500.15)	
Working hours per week (hours)	52.1(13.00)	
Participant commitment		
Total attendance to the program (days)	8.4(3.88)	
Frequency of calorie intake recorded in the logbook (days)	58.4(43.46)	
Frequency of exercise recorded in the logbook (days)	63.7(45.83)	
**Body image perception**		
Negative affect	29.0(7.22)	
Attractive evaluation	18.2(5.06)	
Physical functionality awareness	16.5(3.22)	
Height dissatisfaction	8.9(3.84)	

^a^doctor, assistant environmental health officer, assistant medical officer, driver, pharmacist staff, doctor, public health assistant, dental staff, laboratory technologist, and healthcare assistant

Table 2 shows that five variables which were gender, income, attendance (days), frequency of calorie intake recorded in the logbook (days) and frequency of exercise recorded in the logbook (days) would be analysed in the multiple logistic regression as their p-values were less than 0.25.

**Table 2:** Factors associated with the success in achieving targeted weight reduction among HCPs in Terengganu using simple logistic regression analysis (*n* = 100).

Variables	Success (*n* = 26)		Failed (*n* = 74)		Crude OR (95% CI)	*p*-value
	Mean (SD)	*n* (%)	Mean (SD)	*n* (%)		
Age	37.5 (6.03)		39.2 (6.50)		−0.042 (0.89,1.03)	0.258
Gender						
Male		1 (3.8)		16 (21.6)	1	
Female		25 (96.2)		58 (78.4)	6.90(0.87,54.87)	0.068^*^
Marital status						
Single		3 (11.5)		13 (17.6)	1	
Married		23 (88.5)		61 (82.4)	1.63 (0.43,6.26)	0.474
Highest Education						
Secondary		11 (42.3)		26 (35.1)	1	
Tertiary		15 (57.7)		48 (64.9)	0.74 (0.30,1.84)	0.515
Job position						
Administrative						
Staff		4 (15.4)		9 (12.2)	1	
Nurses		11 (42.3)		33 (44.6)	0.75 (0.19,2.93)	0.679
Other professions^a^		11 (42.3)		32 (43.2)	0.77 (0.20,3.03)	0.712
Income (per RM100)	32.7 (13.01)		42.2 (51.79)		0.98 (0.94,1.01)	0.201^*^
Working hours per week	51.2 (12.21)		52.4 (13.32)		0.99 (0.96,1.03)	0.671
Total attendance to the program (days)	12.5 (2.85)		6.9 (3.11)		1.68 (1.36,2.07)	<0.01^*^
Frequency of calorie intake recorded (days)	107.0 (30.09)		41.3 (33.39)		1.01 (1.04,1.09)	<0.01^*^
Frequency of exercise recorded (days)	113.8 (25.73)		46.1 (37.59)		1.06 (1.03,1.08)	<0.01^*^
Body image perception						
Negative affect	28.4 (7.06)		29.2 (7.31)		0.99 (0.93,1.05)	0.624
Attractive Evaluation	17.9 (4.21)		18.3 (5.35)		0.98 (0.90,1.07)	0.702
Physical Functionality Awareness	16.5 (3.08)		16.5 (3.29)		1.01 (0.88,1.16)	0.914
Height Dissatisfaction	9.5 (3.49)		8.6 (3.95)		1.07 (0.95,1.20)	0.295

^a^doctor, assistant environmental health officer, assistant medical officer, driver, pharmacist staff, doctor, public health assistant, dental staff, laboratory technologist, and healthcare assistant.

* p-value of < 0.25.

Table 3 shows factors associated with the success of targeted weight reduction among HCPs using multiple logistic regression analysis. The significant variables (p-value < 0.05) were income, total attendance to the program, and frequency of recorded calorie intake in the logbook (days). An increase of RM100 in income was linked to a 6.0% decrease in the odds of achieving success in targeted weight reduction [(OR-1) *100] while controlling for the other variables in the model. After controlling for other variables in the model, each additional day of attendance to the program results in approximately a 33.5% increase in the odds of achieving success in targeted weight reduction. Similarly, controlling for other variables in the model, a one-day increase in the frequency of calorie intake recorded in the logbook was associated with a 5% increase in the odds of success in targeted weight reduction.

**Table 3:** Factors associated with the success in achieving targeted weight reduction among healthcare providers in Terengganu using multiple logistic regression analysis (*n* = 100).

Variables	*b*	Adjusted OR (95% CI)	*p*-value
Income (per RM100)	−0.065	0.94 (0.88,1.00)	0.039
Total of attendance to the program (days)^a^	0.289	1.34 (1.02,1.75)	0.035
Frequency of recorded calorie intake in the logbook (days)	0.049	1.05 (1.02,1.08)	0.001

The forward LR method was applied. No multicollinearity and no interaction. Hosmer- Lemeshow test, *p*-value 0.885. Classification table 89.0% correctly classified. The ROC Area Under the Curve was 94.9%.

## Discussion

This study aimed to evaluate the effectiveness of a structured 6-month weight management intervention among healthcare providers (HCPs) and to identify key predictors of weight loss success. The findings revealed that commitment to dietary self-monitoring, regular exercise, and program attendance were significant factors associated with achieving ≥5% weight loss.

The present study found 26.0% of participants successfully achieved the targeted weight reduction during the six-month period. In contrast, a three-month intervention study involving hospital employees reported a significantly higher success rate, with 46.2% of participants achieving weight loss [28]. Similarly, a recent study conducted in Malaysia, which included a three-month group education program combined with dietary counselling from a nutritionist, reported a success rate of 42.0% among participants [27].

It is important to note that both studies employed shorter intervention durations compared to ours. These differences underscore the variability in weight reduction outcomes across studies, suggesting that the length of the intervention may be a critical influencing factor. Research indicates that short-term interventions often yield more immediate positive effects than those lasting longer than six months [36]. However, longer interventions may promote greater sustainability of weight loss beyond the intervention period [37].

Weight loss intervention durations typically range from 3 to 12 months, depending on program intensity and structure. Short-term interventions (≤3 months) often yield rapid weight loss but face challenges in sustaining long-term adherence. For example, a study by Antoni et al. (2018) demonstrated that time-restricted feeding (e.g., an 8-hour feeding window) and calorie-dense meals during breakfast and lunch resulted in significant weight loss (1.4–1.8 kg) over 12 weeks, highlighting the potential of shorter interventions to achieve measurable outcomes [38]. Long-term weight maintenance remains a significant challenge, as research indicates that over 50% of lost weight is regained within two years. This is partly due to biological adaptations, such as changes in fat cell DNA and hormonal regulation, which make regaining weight easier [39].

Sustained weight loss requires long-term behavioural strategies, including regular resistance training, a nutrient-dense diet rich in fibre, fruits, and vegetables, and ongoing lifestyle modifications to regulate appetite hormones and metabolism [40]. Future research should focus on post-intervention follow-ups to assess weight maintenance strategies and identify biological and behavioural factors that contribute to weight regain. Understanding these mechanisms will help develop more effective, long-term weight management programs.

We found that there was an inverse relationship between an increase of RM100 in income and the likelihood of achieving successful targeted weight reduction. This finding contrasts with research conducted in the United States, which indicated that lower-income individuals tend to exhibit lower engagement levels and achieve less favourable outcomes in online behavioural weight loss programs [14]. Conversely, a study by Kruger et al. (2006) found no significant association between income and the success of targeted weight loss [26]. The observed relationship between a RM100 increase in income and a higher probability of failing to achieve weight loss goals may be linked to changes in dietary habits and lifestyle that accompany increased income. According to the Emotional eating, depressive symptoms, and weight loss maintenance. Malaysian food barometer, over 64.0% of the population consumes at least one meal outside of their homes each day [41]. This trend is influenced by factors such as work commitments and the prevalence of dual-income families. Additionally, approximately one-third of the population tends to indulge in heavy meals late at night [41]. In essence, the increase in income may lead to a greater tendency to dine out and consume less healthy foods, which in turn, could lead to weight gain and hinder efforts to achieve targeted weight reduction. Higher-income individuals may also have longer working hours, which could limit their time for meal preparation and physical activity. Additionally, the availability of natural and minimally processed foods in the workplace may be limited, further complicating adherence to healthy eating habits [42].

When adjusting for other variables in the model, each additional day of attendance in the program corresponds to about 34% higher likelihood of achieving the targeted weight reduction. These findings are supported by a study in Korea which found that higher attendance levels were generally associated with a more substantial weight reduction [43]. Similarly, Piemas et al (2020) who conducted a study found the importance of program attendance for the success of the program [13]. Participants who attended more program sessions experienced a significantly greater weight reduction. Regular attendance could help HCPs stay responsible for their weight loss goal. Knowing that they are expected to attend sessions can motivate participants to stay on track with their diet and exercise plans. It creates a sense of responsibility and commitment to their weight loss journey [44].

It was also observed that a one-day increase in the frequency of calorie intake recorded in the logbook was associated with about a 5% increase in the odds of achieving the targeted weight reduction. This finding is in line with prior research which found that consistent tracking using food tracking booklets shows a significant predictor of average weight change in the program [25]. Furthermore, a study found that frequent use of mobile apps to monitor diet and calorie intake, especially focusing on dinner input, played a crucial role in successful weight reduction [23]. Similarly, it was also reported that participants who reported to plan their meals and diligently tracking their calorie intake had a higher percentage of successful weight loss compared to those who did not [26]. Calorie tracking in a logbook may enhance dietary awareness, leading to better decision-making and portion control. Consistent tracking of calorie intake can lead to better food choices and healthier eating habits [45].

From a theoretical perspective, our results are also in align with the self-determination theory, which emphasizes the role of autonomy, competence, and relatedness in behaviour change. Participants who actively engaged in weight reduction programs and consistently monitored their calorie intake demonstrated higher levels of self-regulation and commitment, which are critical for achieving weight loss goals [46].

Many studies highlighted the significant role of body perception in weight management. A study among college students in Malaysia revealed that about 54% of participants accurately perceived their weight based on their actual BMI [47]. Among them, about half made efforts to lose weight. Another study among ≥ 55-year-old adults demonstrated that an individual’s perception of their weight status had a notable influence on their weight management behaviours [48]. Those who believed they were overweight were more likely to engage in weight management activities, such as dieting and exercising, underscoring the motivating role of self-perception in weight control efforts. Conversely, individuals experiencing misperception and dissatisfaction with their body weight tended to make unhealthy lifestyle choices, including engaging in unhealthy behaviours and struggling to adopt healthier habits. Nonetheless, the current study did not find an association between body image perception and the achievement of targeted weight reduction. This discrepancy could be attributed to the distinct characteristics of the population under investigation. College students, frequently influenced by societal ideals of beauty, may hold varying perspectives on body image and weight control [47]. Whereas older individuals prioritise health over appearance, while healthcare workers may have distinct career-related factors affecting their perception.

The predominance of women in our study (83.0%) may have influenced the results, as gender differences in adherence to lifestyle changes are well-documented. Women are generally more likely to engage in weight loss programs and adhere to dietary and exercise recommendations, possibly due to greater health consciousness and social support networks [1,49]. However, in our study, gender was not significantly associated with the success of targeted weight reduction (p = 0.068). This could be due to the small proportion of male participants (17.0%), which may have limited our ability to detect significant gender-related differences. Future studies with a more balanced gender distribution are needed to explore this further.

While the mean age of participants was 38.8 years (SD 6.40), the age range in our study was relatively narrow. Older individuals may face greater challenges in adopting lifestyle changes due to factors such as slower metabolism, chronic health conditions, and established habits [22]. However, our analysis did not reveal a significant association between age and the success of targeted weight reduction. This could be because our sample predominantly consisted of middle-aged adults, and the age range may not have been wide enough to capture significant differences. Future studies should include a broader age range to better understand the impact of age on weight loss outcomes.

Although not statistically significant, our results showed that 88.5% of successful participants were married, compared to 82.4% of those who did not achieve the targeted weight reduction. This aligns with previous research suggesting that married individuals may benefit from social support and shared responsibilities, which can facilitate adherence to lifestyle changes [1]. For example, married individuals may have partners who encourage healthy eating and exercise habits, or they may have more structured routines that support weight management.

The present study found that job position was not significantly associated with the success of targeted weight loss among the healthcare providers after a six-month intervention program. This could be because healthcare providers, regardless of their specific roles, often share similar work-related challenges, such as high stress, long hours, and irregular schedules, which may uniformly influence their ability to adhere to weight loss programs [50]. Additionally, the design of the intervention might have been equally effective across all job positions, minimizing the impact of role-specific differences [51]. Individual factors like motivation, baseline health, and personal habits could also play a stronger role in weight loss success than job-related factors [52]. Additionally, job position is a broad category and may not capture the specific daily routines or stressors that truly impact weight loss outcomes [53].

Among the limitations of our study was a small sample size. Although the required sample size was 121, only 100 participants met the inclusion criteria. To minimize bias, we applied multivariable analysis and imputed missing data using the Last Observation Carried Forward (LOCF) method. Additionally, the study relied on self-reported data for exercise frequency, calorie intake, and exercise documented in logbooks. Self-reports are prone to recall bias, as participants may inaccurately remember or estimate their behaviours. Additionally, social desirability bias may lead participants to under-report unhealthy behaviours such as overeating or skipping exercise and over-report healthier habits to present themselves in a more favourable light [54]. These biases can compromise the accuracy of the data and limit the validity of the findings.

Validating self-reported data is a significant challenge in research. Traditional methods, such as paper-based logbooks, are often incomplete or inconsistently maintained. Participants may forget to record entries or provide vague descriptions of their dietary intake and physical activity. This lack of precision makes it difficult for us to draw reliable conclusions about participants’ actual behaviours [55].

To address these challenges, we suggest future researchers to use online logbook applications for dietary and exercise tracking. These tools allow participants to record their food consumption and physical activity in real-time, reducing reliance on memory and minimizing recall bias. On top of that, nutritionists can access and analyze the data more effectively, ensuring greater accuracy and reliability. For example, apps like MyFitnessPal or Fitbit have been shown to improve the precision of self-reported data by providing prompts, reminders, and detailed tracking features [56].

While online logbook applications offer significant advantages, they are not without challenges. Not all participants may be comfortable or proficient with technology, which could lead to incomplete or inconsistent data entry. Additionally, the cost and accessibility of such tools may limit their use in certain populations. Researchers must also ensure that the apps used are user-friendly and tailored to the study population to maximize compliance and data quality [57].

Despite these challenges, the integration of technology represents a significant advance in research methodology. Real-time data collection tools not only improve the accuracy of self-reported information but also enable researchers to monitor participant adherence more closely. This can lead to more robust findings and a better understanding of the factors influencing weight loss success. Furthermore, combining self-reported data with objective measures such as wearable devices for physical activity or biomarkers for dietary intake can provide a more comprehensive picture of participants’ behaviours [58].

Healthcare professionals often face significant challenges, including high stress, long working hours, and limited access to wellness programs. This lack of support can lead to burnout, reduced job satisfaction, and poor mental and physical health. Studies have shown that when healthcare workers are not adequately cared for, their ability to provide high-quality patient care is compromised [53]. The well-being of healthcare professionals is directly linked to the quality of care they provide. For example, burned-out or overweight healthcare workers may be less effective in delivering patient care, making errors more likely, and reducing patient satisfaction [59]. This not only affects individual patient outcomes but also has broader implications for public health, as the overall efficiency and effectiveness of healthcare systems depend on the health and performance of their workforce [60]. To address these issues, systemic changes are needed to prioritize the well-being of healthcare professionals. This includes implementing workplace wellness programs, providing access to mental health resources, and creating supportive environments that encourage healthy lifestyles. By improving the quality of life for healthcare workers, we can enhance the quality of care they deliver and, ultimately, improve public health outcomes [61].

## Conclusions

The results of this study underscore the importance of active participation in weight reduction programs, and consistent calorie monitoring in achieving targeted weight loss among healthcare providers. Participants with lower income levels may have been less likely to dine out, potentially contributing to their success in reaching weight reduction goals. Furthermore, active involvement in structured weight loss programs significantly increased the likelihood of achieving desired outcomes.

Our findings highlight the need for targeted interventions to support the well-being of healthcare providers. By addressing the fragility in care provided to these workers, we can not only improve their quality of life but also enhance the quality of care they deliver, ultimately benefiting public health. Future research should explore systemic changes and workplace interventions that prioritize the health and well-being of healthcare providers.
